# fMRI data of mixed gambles from the Neuroimaging Analysis Replication and Prediction Study

**DOI:** 10.1038/s41597-019-0113-7

**Published:** 2019-07-01

**Authors:** Rotem Botvinik-Nezer, Roni Iwanir, Felix Holzmeister, Jürgen Huber, Magnus Johannesson, Michael Kirchler, Anna Dreber, Colin F. Camerer, Russell A. Poldrack, Tom Schonberg

**Affiliations:** 1 Sagol School of Neuroscience, Tel Aviv University, Tel Aviv-Yafo, Israel; 2 Faculty of Life Sciences, Department of Neurobiology, Tel Aviv University, Tel Aviv-Yafo, Israel; 3 Department of Banking and Finance, University of Innsbruck, Innsbruck, Austria; 4 Department of Economics, Stockholm School of Economics, Stockholm, Sweden; 5 Department of Economics, University of Innsbruck, Innsbruck, Austria; 6 Division of Humanities and Social Sciences, California Institute of Technology, Pasadena, CA USA; 7 Department of Psychology, Stanford University, Stanford, CA USA

**Keywords:** Human behaviour, Reward, Functional magnetic resonance imaging, Brain imaging, Decision

## Abstract

There is an ongoing debate about the replicability of neuroimaging research. It was suggested that one of the main reasons for the high rate of false positive results is the many degrees of freedom researchers have during data analysis. In the Neuroimaging Analysis Replication and Prediction Study (NARPS), we aim to provide the first scientific evidence on the variability of results across analysis teams in neuroscience. We collected fMRI data from 108 participants during two versions of the mixed gambles task, which is often used to study decision-making under risk. For each participant, the dataset includes an anatomical (T1 weighted) scan and fMRI as well as behavioral data from four runs of the task. The dataset is shared through OpenNeuro and is formatted according to the Brain Imaging Data Structure (BIDS) standard. Data pre-processed with fMRIprep and quality control reports are also publicly shared. This dataset can be used to study decision-making under risk and to test replicability and interpretability of previous results in the field.

## Abstract (summary)

**Table:** 

Design Type(s)	data integration objective • data validation objective • behavioral data analysis objective
Measurement Type(s)	brain activity measurement
Technology Type(s)	magnetic resonance imaging
Factor Type(s)	sex • experimental condition • age
Sample Characteristic(s)	Homo sapiens • brain

Machine-accessible metadata file describing the reported data (ISA-Tab format)

## Background & Summary

The recent “replication crisis” in many scientific fields has raised broad concern regarding the reliability of published results^1–3^. One main reason for the high rate of false positive results is the large number of “researcher degrees of freedom”, wherein the process of data analysis can be modified towards desired results based on data itself^4–6^. There is an increasing debate regarding the replicability of research in general and specifically of neuroimaging, which has a thriving “garden of forking analysis paths”^7,8^.

In the Neuroimaging Analysis Replication and Prediction Study (NARPS; https://www.narps.info/), we collected fMRI data in order to estimate the variability of neuroimaging results across analysis teams, and measure peer beliefs about the results by running prediction markets^3,9^. Dozens of teams were given the data to independently analyze it according to pre-defined hypotheses. For this study we chose to examine the neural basis of decision-making under risk, which is defined as a choice between options that yield different known outcomes with known probabilities^10,11^. The mixed gambles task, which we used in NARPS, is frequently used to study risky decision-making. On each trial, participants are asked to accept or reject a prospect consisting of an equal 50% chance of either gaining or losing some (equal or different) amount of money.

The most accepted theory of risky decision-making is prospect theory^12^, which suggests that people make risky decisions based on subjective values of potential losses and gains, compared to a reference point, as well as individual differences in risk preferences^12^. One prominent phenomenon is loss aversion: people tend to be more sensitive to losses compared to equal-sized gains^13^. According to previous studies, people will typically avoid gambles in which the potential gain is less than twice the potential loss^13–16^ (but see Gal and Rucker^17^).

A fundamental question in the field is whether potential losses elicit negative emotions, which drive loss aversion, or rather the same neural systems, encoding subjective value, asymmetrically respond to losses compared to gains. Tom *et al*.^18^ studied the neural basis of loss aversion with the mixed gambles task. They found that fMRI activity in many regions (including dopaminergic midbrain regions and their targets) increased as potential gains increased, while activity in most of these regions decreased as potential losses increased. No regions showed significant stronger fMRI activity for larger potential losses, even when tested at a lower threshold. When focusing on the ventromedial prefrontal cortex and striatum, they further revealed a greater slope for the losses-related activity compared to the gains-related activity (referred to as “neural loss aversion”). These results were interpreted as showing that gains and losses are encoded by the same neural system and loss aversion is related to greater neural sensitivity to potential losses compared to potential gains, rather than to engagement of systems related to negative emotion or aversion.

Other work had suggested that regions related to negative emotion are involved in loss aversion. De Martino *et al*.^19^ tested two individuals with bilateral amygdala lesions compared to healthy matched controls. They found that both amygdala-lesioned participants were not loss averse, while their match-controls were. Canessa *et al*.^20^ aimed to resolve the inconsistent previous results by focusing on individual differences in loss aversion. They found both bidirectional as well as gain/loss-specific fMRI activity during choices. This difference could reflect the relatively low power of the Tom *et al*. study, but could also reflect a difference in the design between the two studies. The Tom *et al*. study used a design in which the potential losses were half of the potential gains (“equal indifference”), in order that roughly half of the gambles would be accepted (given a loss aversion ratio of ~2), whereas in the studies of De Martino *et al*. and Canessa *et al*., the range of gains was equal to the range of losses (“equal range”).

We share behavioral, anatomical and fMRI data from 108 healthy participants, each performing one version of the mixed gambles task (equal range/equal indifference). This dataset was collected in order to test replicability and resolve inconsistencies of previous results. Moreover, the two conditions used provide a unique opportunity to compare the interactions between the specific gains/losses matrix used and the elicited behavior and neural fMRI activity.

## Methods

### Participants

N = 119 healthy participants completed the experiment (n = 60 from the equal indifference condition^18^ and n = 59 from the equal range condition^19^). Nine participants were excluded prior to fMRI analysis based on preregistered exclusion criteria (https://aspredicted.org/ru8ua.pdf): Five did not show a significant effect of both gains and losses on their choices (Bayesian logistic regression, with gains and losses as independent regressors and acceptance or rejection of the gamble as the dependent variable; excluded participants had p > 0.05 for either gains, losses or both, reflecting a lack of understanding of the task) and four missed over 10% of trials (in one or more runs). Preprocessing of two additional participants failed due to memory errors, which could not be resolved within the time frame of the study. These two participants were therefore excluded as well. Thus, 108 participants (Equal indifference condition: n = 54, 30 females, mean age = 26.06 ± 3.02 years; equal range condition: n = 54, 30 females, mean age = 25.04 ± 3.99 years) are included in the final dataset shared on OpenNeuro^21^. Demographic information for all participants (including gender and age) can be found in the participants.tsv file on OpenNeuro.

All participants were right-handed, had normal or corrected-to-normal vision and reported no history of psychiatric or neurologic diagnoses, or use any medications that would interfere with the experiment. The experiment lasted about two hours, out of which ~70 minutes were performed inside the MRI scanner. Participants were paid 120 ILS (about 32$ USD), plus/minus the amount of money they gained/lost, respectively, based on the mixed gambles task (ranging between −20 and +40 ILS). All participants gave written informed consent. The study was approved by the institutional review board at the Sheba Tel Hashomer Medical Center and the ethics committee at Tel Aviv University.

### Experimental procedures

When arriving to the lab, participants signed the consent forms. Then, they were endowed with 20 ILS in cash. The experimenter explained that the money is theirs to keep, and is part of the full payment they will receive at the end of the experiment. Next, participants were given general instructions regarding behavior inside the scanner and performed a shortened version of the full task (i.e. demo) in a behavioral testing room located at the imaging center at Tel Aviv University. After the demo, the experimenter asked the participant whether s/he understood the task.

Following these preparations, participants entered the MRI scanner. They completed four runs of the mixed gambles task while being scanned with fMRI. Then, we acquired field mapping and anatomical scans while participants rested inside the scanner (i.e. no task was performed during these scans).

Participants also underwent scanning of other sequences not shared in the present dataset release: Resting state fMRI data were acquired during 10 minutes in which participants were asked to stay awake and rest with their eyes open while a fixation point was presented on a grey screen. In the final part inside the scanner, participants were scanned with diffusion-weighted imaging (DWI; the first 18 participants, as well as other participants, were not scanned with DWI). Eye gaze data were collected during the fMRI scans using an EyeLink 1000 Plus SR-Research eye-tracker. Finally, participants returned to the behavioral testing room, where they completed the five following questionnaires: brief self-control index^22^, domain-specific risk-taking (DOSPERT)^23^, ten-item personality (TIPI)^24^, risk aversion (Holt Laury questionnaire)^25^ and delay discounting (monetary-choice questionnaire)^26^. These questionnaires are also not included as part of the present dataset release. The experiment ended with a lottery in which one gamble from the mixed gambles task was randomly chosen and played (if it had been accepted).

### Mixed gambles task

**Fig. 1:**
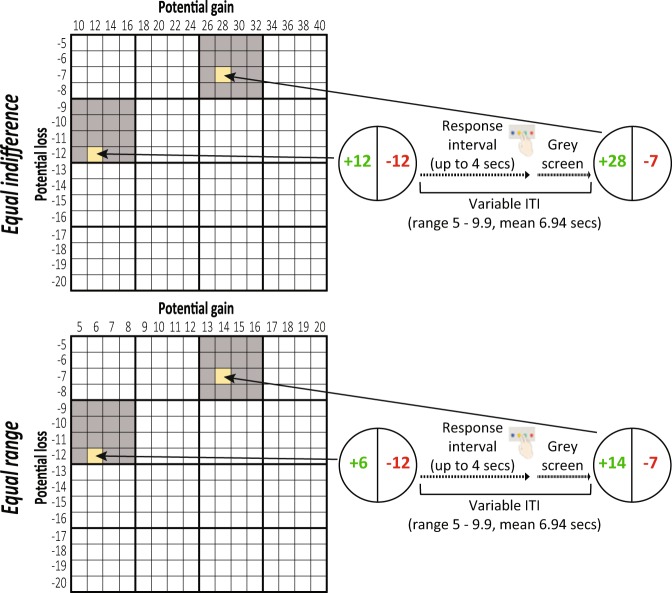
An illustration of the mixed gambles task design. Adapted from Tom *et al*.^18^. During each trial, the participant was presented with prospects including the potential gain and loss, until a response was made or four seconds passed. The next gamble was presented following a jittered inter-trial interval (ITI). Potential gains and losses were sampled from the presented gain/loss matrices, which were different for the equal indifference and equal range conditions. The amounts of money are in ILS.

Each participant completed four runs of the mixed gambles task. Each run consisted of 64 trials performed during an fMRI scanning run lasting 453 seconds and comprising 453 volumes (given the repetition time of one second) see Fig. 1.

On each trial, participants were presented with a mixed gamble entailing an equal 50% chance of gaining one amount of money or losing another amount. Possible gains ranged between 10 and 40 ILS (in increments of 2 ILS) in the equal indifference condition^18^ or 5–20 ILS (in increments of 1 ILS) in the equal range condition^19^, while possible losses ranged from 5–20 ILS (in increments of 1 ILS) in both conditions. All 256 possible combinations of gains and losses were presented to each participant across the four runs of the task.

Similar to previous experiments^18^, participants were asked to decide within four seconds whether or not they would like to accept each of the gambles presented to them, with four possible responses: strongly accept, weakly accept, weakly reject or strongly reject. Participants responded with four fingers (all fingers except the thumb) on an MRI-compatible response box located in their right hand. In order to elicit incentive-compatible behavior, participants were endowed with 20 ILS at the beginning of the experiment, and were told in advance that one trial would be randomly selected at the end of the experiment and if they had accepted (i.e. responded with either “strongly accept” or “weakly accept”) that gamble during the task, the gamble would be played for real money.

Stimulus presentation was similar to Tom *et al*.^18^. The stimulus consisted of a circle presented on a gray (RGB = [127, 127, 127]) screen and divided to two halves: on one side the gain amount was presented in green (RGB = [0, 210, 0]) with a ‘plus’ sign before the number, and on the other side the loss amount was presented in red (RGB = [240, 0, 0]) with a ‘minus’ sign before the number (see Fig. 1). The allocation of the gain/loss amounts to the left/right side was consistent for each participant, but counter-balanced across participants. The gamble was presented on the screen until the participant responded or four seconds have passed, followed by a grey screen until the onset of the next trial.

The experiment was computed using Matlab version 2014b and the Psychtoolbox (www.psychtoolbox.org). The timing and order of stimulus presentation was optimized for efficiency using a tailored code from the creators of neuropowertools (http://neuropowertools.org/). The efficiency calculations assumed a 32 seconds HRF, a one second TR and a truncated exponential distribution of inter-trial intervals (ITIs; min = 5 seconds, max = 10 seconds, mean = 7 seconds; Lambda was extrapolated from these parameters). The minimal ITI encompassed a potential trial duration of four seconds and a break of one second. In order to even the gain and loss amounts between the different runs, the full matrix of gambles was divided into 16 4 × 4 sub matrices, which were independently scrambled and allocated to the different runs. This procedure ensured similarity between runs. Using these procedures, we created for each condition (equal indifference/equal range) eight different combinations of gambles and onsets divided to four runs. These matrices are shared with the code in the GitHub repository (https://github.com/rotemb9/NARPS_scientific_data) as one Matlab (.mat) file for each condition (“*equalIndifference_design*.*mat*” and “*equalRange_design*.*mat*”). The specific combination used for each participant was counterbalanced across participants. The final ITIs for each run, in both conditions, ranged from 5 to 9.9 seconds, with an averaged ITI of 6.94 seconds.

### MRI data acquisition

Imaging data were acquired using a 3 T Siemens Prisma MRI scanner with a 64-channel head coil, at the Strauss Imaging Center on the campus of Tel Aviv University. Functional data during the mixed gambles task were acquired using T2*-weighted echo-planar imaging sequence with multi-band acceleration factor of 4 and parallel imaging factor (iPAT) of 2, TR = 1000 ms, TE = 30 ms, flip angle = 68 degrees, field of view (FOV) = 212 × 212 mm, in plane resolution of 2 × 2 mm 30 degrees off the anterior commissure-posterior commissure line to reduce the frontal signal dropout^27^, slice thickness of 2 mm, 64 slices and a gap of 0.4 mm between slices to cover the entire brain. For each functional run, we acquired 453 volumes.

We also acquired gradient echo field maps for each participant, with the following imaging parameters: TR = 400 ms, TE 1 = 4.92 ms, TE 2 = 7.38 ms, flip angle = 60 degrees, FOV = 212 × 212 mm, in plane resolution of 2 × 2 mm, 33 slices with slice thickness of 4 mm and a gap of 0.8 mm between slices. In addition, for each participant we acquired high-resolution T1 weighted structural images using a magnetization prepared rapid gradient echo (MPRAGE) pulse sequence with parallel imaging factor (iPAT) of 2, TR = 2530 ms, TE = 2.99 ms, flip angle = 7 degrees, FOV = 224 × 224 mm, resolution = 1 × 1 × 1 mm.

### MRI pre-processing

The pre-processed data included in this dataset were preprocessed using fMRIprep version 1.1.4^28^, which is based on Nipype 1.1.1^29,30^.

### Anatomical data preprocessing

Each of the T1-weighted (T1w) images were preprocessed with the following pipeline: First, the T1w image was corrected for intensity non-uniformity (INU) using N4BiasFieldCorrection^31^ (ANTs 2.2.0), and used as T1w-reference throughout the workflow. The T1w-reference was then skull-stripped using antsBrainExtraction.sh (ANTs 2.2.0), with OASIS as target template. Brain surfaces were reconstructed using recon-all (FreeSurfer 6.0.1 RRID:SCR_001847)^32^, and the brain mask estimated previously was refined with a custom variation of the method to reconcile ANTs-derived and FreeSurfer-derived segmentations of the cortical gray-matter of Mindboggle (RRID:SCR_002438)^33^. Spatial normalization to the ICBM 152 Nonlinear Asymmetrical template version 2009c (RRID:SCR_008796)^34^ was performed through nonlinear registration with antsRegistration (ANTs 2.2.0, RRID:SCR_004757)^35^, using brain-extracted versions of both T1w volume and template. Brain tissue segmentation of cerebrospinal fluid (CSF), white-matter (WM) and gray-matter (GM) was performed on the brain-extracted T1w using fast (FSL 5.0.9, RRID:SCR_002823)^36^.

### Functional data preprocessing

For each of the four BOLD runs per participant, the following preprocessing was performed: First, a reference volume and its skull-stripped version were generated using a custom methodology of fMRIPrep. A deformation field to correct for susceptibility distortions was estimated based on a field map that was co-registered to the BOLD reference, using a custom workflow of fMRIPrep derived from D. Greve’s epidewarp.fsl script and further improvements of HCP Pipelines^37^. Based on the estimated susceptibility distortion, an unwarped BOLD reference was calculated for a more accurate co-registration with the anatomical reference. Head-motion parameters with respect to the BOLD reference (transformation matrices, and six corresponding rotation and translation parameters) are estimated before any spatiotemporal filtering using mcflirt (FSL 5.0.9)^38^. The BOLD time-series (including slice-timing correction when applied) were resampled onto their original, native space by applying a single, composite transform to correct for head-motion and susceptibility distortions. These resampled BOLD time-series will be referred to as preprocessed BOLD in original space, or just preprocessed BOLD. The BOLD reference was then co-registered to the T1w reference using bbregister (FreeSurfer) which implements boundary-based registration^39^. Co-registration was configured with nine degrees of freedom to account for distortions remaining in the BOLD reference. The BOLD time-series, were resampled to surfaces on the following spaces: fsaverage5. The BOLD time-series were resampled to MNI152NLin2009cAsym standard space, generating a preprocessed BOLD run in MNI152NLin2009cAsym space. Several confounding time-series were calculated based on the preprocessed BOLD: framewise displacement (FD), DVARS and three region-wise global signals. FD and DVARS are calculated for each functional run, both using their implementations in Nipype (following the definitions by Power *et al*.^40^). The three global signals are extracted within the CSF, the WM, and the whole-brain masks. Additionally, a set of physiological regressors were extracted to allow for component-based noise correction (CompCor^41^). Principal components are estimated after high-pass filtering the preprocessed BOLD time-series (using a discrete cosine filter with 128 s cut-off) for the two CompCor variants: temporal (tCompCor) and anatomical (aCompCor). Six tCompCor components are then calculated from the top 5% variable voxels within a mask covering the subcortical regions. This subcortical mask is obtained by heavily eroding the brain mask, which ensures it does not include cortical GM regions. For aCompCor, six components are calculated within the intersection of the aforementioned mask and the union of CSF and WM masks calculated in T1w space, after their projection to the native space of each functional run (using the inverse BOLD-to-T1w transformation). The head-motion estimates calculated in the correction step were also placed within the corresponding confounds file. All resamplings can be performed with a single interpolation step by composing all the pertinent transformations (i.e. head-motion transform matrices, susceptibility distortion correction when available, and co-registrations to anatomical and template spaces). Gridded (volumetric) resamplings were performed using antsApplyTransforms (ANTs), configured with Lanczos interpolation to minimize the smoothing effects of other kernels^42^. Non-gridded (surface) resamplings were performed using mri_vol2surf (FreeSurfer).

Many internal operations of fMRIprep use Nilearn 0.4.2^43^ (RRID:SCR_001362), mostly within the functional processing workflow. For more details of the pipeline, see the section corresponding to workflows in fMRIPrep’s documentation (https://fmriprep.readthedocs.io/en/1.1.4/workflows.html).

## Data Records

This dataset is organized according to the Brain Imaging Data Structure (BIDS) specification^44^ (version 1.0.1). Full description and documentation of BIDS, including the directories structure, naming conventions and metadata files can be found at http://bids.neuroimaging.io/. Shortly, the data of each participant can be found under the directory “sub-<id>” (e.g. sub-001), with sub-directories for the functional (and behavioral; “func”), field map (“fmap”) and structural (“anat”) data. Metadata is provided in json files. In addition to the raw data, the dataset also include data pre-processed with fMRIprep^28^ version 1.1.4^28^ (https://fmriprep.readthedocs.io/en/1.1.4/usage.html), which can be found under the “derivatives/fmriprep” directory, as well as quality assessment reports generated with MRIQC 0.14.2^45^ (https://mriqc.readthedocs.io/en/0.14.2/), which can be found under the “derivatives/MRIQC” directory.

The NARPS dataset is openly available via the OpenNeuro repository^21^.

## Technical Validation

### Behavioral data validation

We used preregistered criteria (https://aspredicted.org/ru8ua.pdf) to exclude participants that did not demonstrate engagement and comprehension throughout the task. For example, we excluded participants whose choices were not affected by the different amounts of gains and losses (i.e. did not fully understand the task or were indifferent to the task) or missed more than 10% of the trials. Exclusions are fully described under the *‘participants’* sub-section of the *methods* section.

**Fig. 2:**
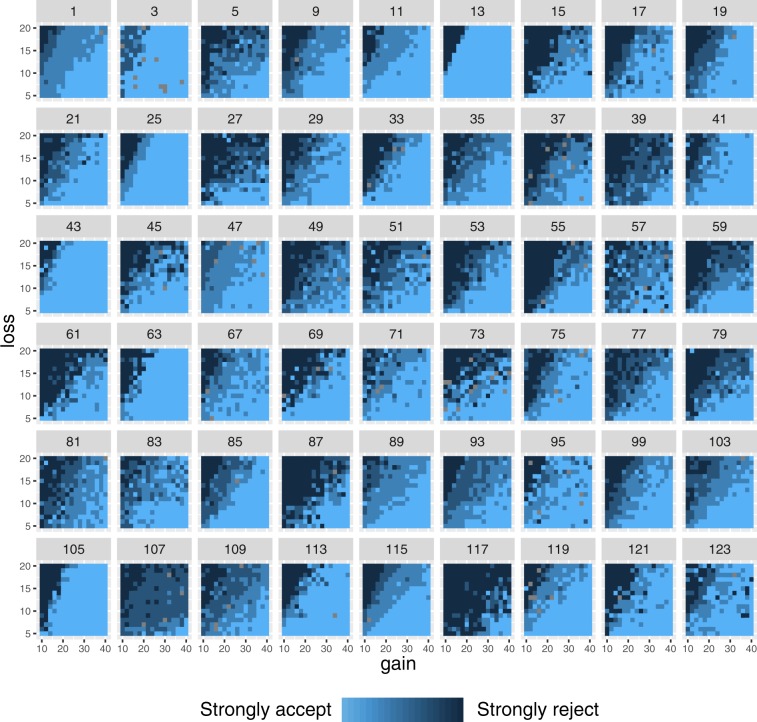
Response matrices for all participants, equal indifference condition. For each participant, the matrix represents the response (strongly accept/weakly accept/weakly reject/strongly reject) for each combination of gain (x axis) and loss (y axis) values.

**Fig. 3:**
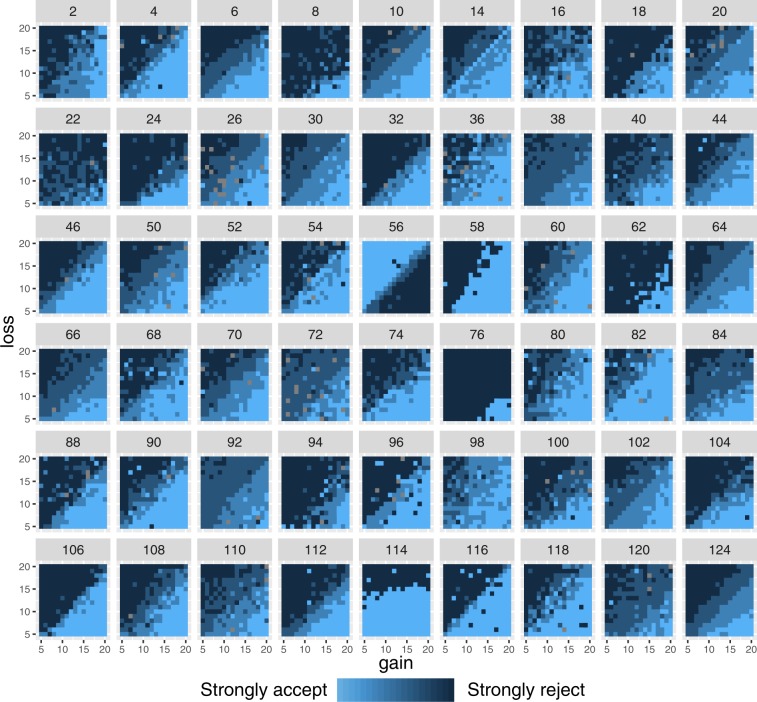
Response matrices for all participants, equal range condition. For each participant, the matrix represents the response (strongly accept/weakly accept/weakly reject/strongly reject) for each combination of gain (x axis) and loss (y axis) values.

Furthermore, we performed basic analysis on the behavioral data, which can be used by users of the dataset. Code for this analysis is available on GitHub (https://github.com/rotemb9/NARPS_scientific_data). Across the entire dataset, that included 27,648 trials (256 trials for each of the 108 participants), there were 194 trials (0.7%) in which participants did not respond in time (mean number of missed trials per participant across the entire task = 1.796 out of 256 trials, SD = 2.834, range = 0–16). These trials are marked as “NoResp” in the behavioral tsv files included in our dataset, and were assigned a response time (RT) value of 0. In addition, we created heat maps for the responses of each participant across the gain/loss matrix (see Fig. 2 for the matrices of the equal indifference condition and Fig. 3 for the matrices of the equal range condition). Based on these heat maps, it seems that sub-056 had reversed the accept/reject responses. This was not one of our preregistered exclusion criteria and thus this participant is included in the dataset.

### Imaging data validation

#### Quality assessment

Quality assessment of large MRI datasets is challenging^45^. Visual inspection, which was the popular method for quality control in previous years, has two main limitations: First, it is highly subjective and requires extensive experience and expertise with MRI data. Second, it is time consuming and impractical for very large datasets, which are becoming more frequent^46,47^.

In order to assess quality of the current dataset, we generated visual quality reports for the anatomical and functional images with MRIQC^45^. MRIQC generates both individual reports for each scan of each participant and group reports to identify outliers. The individual functional reports include for example mosaic view of the average BOLD signal, temporal standard deviation map, plots of motion-related parameters, components resulting from independent component analysis (ICA) and several extracted Image Quality Metrics (IQMs). The individual anatomical (T1w) reports include for example mosaic zoomed-in view of the brain, map of background noise and several extracted IQMs. The group reports visualize the IQMs across participants with a one strip-plot per IQM. This presentation allows relatively easy identification of within-sample outliers. All reports are shared along with the dataset on OpenNeuro, including individual reports for each mixed gambles task run of each participant, individual reports for the anatomical images of each participant, group anatomical report and group functional report. In addition, MRIQC tool also creates a summary JSON files with the IQMs, which are also included in the shared dataset. Based on the group reports generated for the current dataset, several outliers can be identified. For example, in our group BOLD report, the fourth run of one of the participants (sub-036) can clearly be identified as an outlier in the root mean squared (RMS) intensity difference from one volume to the next (DVARS) metric. However, since there is currently no accepted “gold standard” for MRI data quality, we did not exclude participants from the dataset based on image quality assessment (besides the two participants we were unable to preprocess with fMRIprep due to memory errors). Rather, we share the quality assessment reports together with the dataset, in order to allow data users to use the data as they desire, as well as to allow the use of future tools for quality assessment and to apply new standards, once such standards are set.

#### Basic analysis of task versus baseline

In order to validate the data, we further performed basic analysis of fMRI activity during task versus baseline; this contrast is orthogonal to the contrasts of interest in the NARPS project. We used the data preprocessed with fMRIprep that are included in the dataset. Analysis was performed using FEAT (fMRI Expert Analysis Tool) version 6.0.0, part of FMRIB’S Software Library (FSL)^48^.

We used a general linear model (GLM) with three regressors, all of them with the onsets of all trials and the mean RT of the condition (equal indifference/equal range) as duration. The first regressor was without modulation and used to analyze task versus baseline fMRI activity. The other two modelled the demeaned gains and the demeaned losses. These regressors were convolved with the canonical double-gamma hemodynamic response function (HRF), and their temporal derivatives were added to the model. In addition, we added the following nuisance regressors as confounds: standard deviation of DVARS, six aCompCor regressors^41^, framewise displacement and 24 motion regressors (“Friston24”: six motion parameters - translation and rotation in three directions, the square of the six motion parameters and their temporal derivatives). We further modeled out volumes with extensive motion (i.e. scrubbing) by adding a single time-point nuisance regressor for each volume with framewise displacement value greater than 0.9 (an arbitrary threshold meant to serve as a relatively high threshold for motion exclusion, consistent with Siegel *et al*.^49^). One participant (sub-030) was excluded from analysis based on extensive motion, with more than 100 scrubbed volumes for each run. After exclusion of this participant, the mean number of scrubbed volumes from the entire task across participants was 4.08 (range 0–63). In the first level analysis, we estimated a model with the above described regressors for each run of the mixed gambles task of each participant. Each volume was spatially smoothed with FWHM (Full Width at Half Maximum) of 5 mm. In the second level analysis, we averaged the four runs of each participant (fixed effects).

**Fig. 4:**
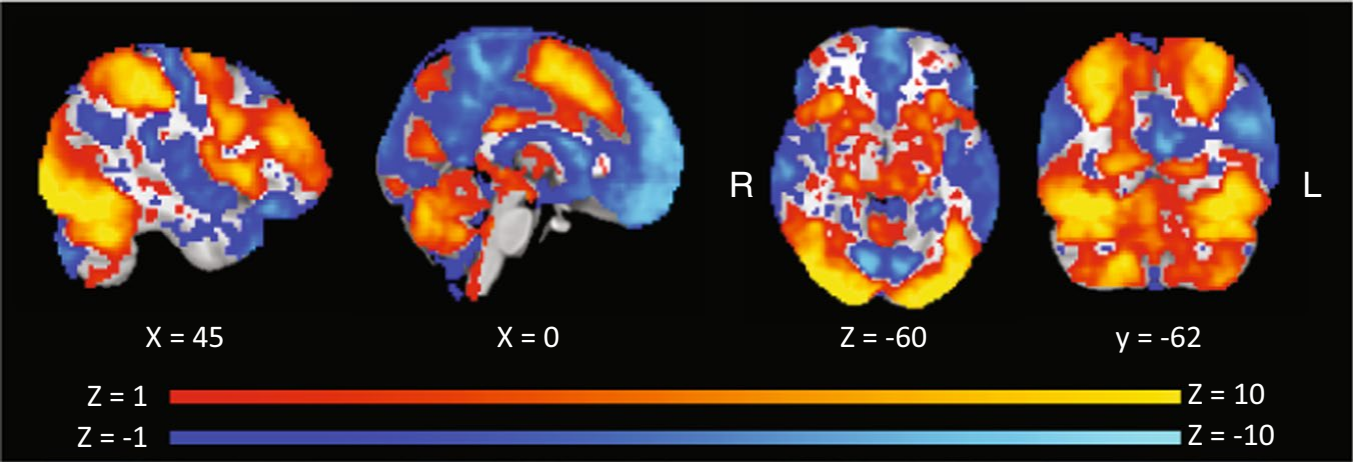
Uncorrected results of task versus baseline. Uncorrected Z values are presented, thresholded at Z > 1 for positive activations (hot colors) and Z < −1 for negative activations (cold colors). This analysis was only used for validation.

Second level analyses of 107 participants were inputted to a group level analysis (mixed effects). This analysis was performed in order to validate the data. Therefore, we only describe the results for the task versus baseline contrast, across the full sample (both conditions). As expected, we found strong positive activity in visual and motor regions and strong negative activity in the default mode network (among other regions). The uncorrected results of this contrast are shown in Fig. 4.

## ISA-Tab metadata file

Download metadata file

## Data Availability

The code for the mixed gambles task were adopted from a previous study^18^. All codes were executed using Matlab version 2014b and the Psychtoolbox (www.psychtoolbox.org). Code is available on GitHub (https://github.com/rotemb9/NARPS_scientific_data). Preprocessed data included in the dataset were preprocessed with fMRIprep version 1.1.4^28^, available from fmriprep.org The quality assessment reports were generated with MRIQC version 0.14.2^45^, available from mriqc.org.
